# Ambient pressure XPS at MAX IV

**DOI:** 10.3762/bjnano.16.118

**Published:** 2025-09-24

**Authors:** Mattia Scardamaglia, Ulrike Küst, Alexander Klyushin, Rosemary Jones, Jan Knudsen, Robert Temperton, Andrey Shavorskiy, Esko Kokkonen

**Affiliations:** 1 MAX IV Laboratory, Lund University, Box 118, SE-221 00, Lund, Sweden; 2 Division of Synchrotron Radiation Research, Lund University, Box 118, SE-221 00, Lund, Sweden; 3 NanoLund, Lund University, Box 118, SE-221 00, Lund, Sweden

**Keywords:** 2D materials, atomic layer deposition, batteries, catalysis, corrosion

## Abstract

Ambient pressure X-ray photoelectron spectroscopy (APXPS) has emerged as an important technique for investigating surface and interface chemistry under realistic conditions, overcoming the limitations of conventional XPS restricted to ultrahigh vacuum. This review highlights the capabilities and scientific impact of APXPS at the MAX IV Laboratory, the world’s first fourth-generation synchrotron light source. With the APXPS beamlines SPECIES and HIPPIE, MAX IV offers state-of-the-art instrumentation for in situ and operando studies across a broad pressure range, enabling research in catalysis, corrosion, energy storage, and thin film growth. The high brilliance and small beam size of MAX IV’s synchrotron light are essential for pushing the time-resolution boundaries of APXPS, especially in the soft X-ray regime. We discuss representative studies at MAX IV, including investigations of single-atom catalysts, confined catalysis, time-resolved catalysis, atomic layer deposition, and electrochemical interfaces, showcasing the role of APXPS in advancing material and surface science.

## Review

### Ambient pressure XPS

Electron spectroscopy has significantly contributed to the understanding of chemical and physical processes that govern the complex interactions between a solid surface and its environment. These processes play crucial roles in phenomena such as heterogeneous catalysis, corrosion, and thin film growth. Given that surfaces are heavily influenced by their surroundings, it is essential to study them in situ, while exposed to realistic reaction conditions, or operando, when producing reaction products under realistic conditions. X-ray photoelectron spectroscopy (XPS) is a powerful surface science technique that enables the investigation of modifications in the chemical environment of a sample surface and its electronic states, owing to its exceptional surface sensitivity. However, the requirement for ultrahigh vacuum (UHV) conditions previously limited the use of XPS in reactive environments. Ambient pressure XPS (APXPS) with soft X-rays addresses this limitation by enabling surface chemical analysis at pressures up to the millibar range, bridging the gap that restricts conventional XPS to UHV. This allows for in situ and operando investigations of material interfaces under more realistic conditions, which is a critical advancement in material research, gaining increasing popularity across various fields. APXPS is crucial for studying dynamic processes in catalysis, environmental science, and energy materials, where reactions typically occur at or near ambient pressure.

Fourth-generation synchrotron light sources, characterized by their unprecedented photon flux, provide a key advantage for APXPS, enabling rapid data acquisition with low signal-to-noise ratio and facilitating the study of transient phenomena. This capability is essential for tracking surface chemical processes in real time.

Due to the scattering of the photoelectrons in a gas environment at millibar pressures, APXPS is intrinsically a photon-hungry technique. Although pioneering work on gases and liquids performed by Kai and Hans Siegbahn date back to the 1970s [1], it was only at the turn of the century that APXPS instruments were developed, thanks to the high flux of the third and then, particularly, fourth generation of synchrotron radiation light sources. At the same time, the development of differentially pumped electron energy analyzers (EEA) with higher transmission also played a crucial role [2]. The technical development of APXPS was driven forward in particular by groups at BESSY II (Berlin, Germany) and ALS (Berkeley, US) [3–4]. Today, APXPS is a consolidated technique, and instruments are widely available both in synchrotron radiation facilities and in university laboratories [5].

MAX IV Laboratory in Lund is a Swedish national laboratory inaugurated in 2016; it was the first fourth-generation synchrotron light source worldwide [6–7]. The accelerator complex comprises a linear accelerator as well as a 1.5 GeV and a 3 GeV storage ring for electrons. MAX IV offers access to 16 beamlines, soon 17; among them, SPECIES and HIPPIE are dedicated to APXPS and are located on the two rings, respectively. Both beamlines offer a portfolio of experimental setups that allow one to explore different aspects of scientific research dedicated to the investigation of solid–gas, solid–liquid, and liquid–gas interfaces in situ and under operando conditions.

In this review, we highlight exemplary APXPS experiments conducted in different environments to study solid–gas and solid–liquid interfaces. APXPS at MAX IV is making a considerable contribution in various scientific fields including catalysis, energy materials, and corrosion, to name a few. As an example in catalysis research, we will discuss experiments on solid–gas interfaces about single-atom catalysts, catalysis in confined space, time-resolved catalysis, and photocatalysis. Remaining in the solid–gas environment, atomic layer deposition (ALD) is another field particularly developed at MAX IV. Also, the ultrahigh brightness of MAX IV ring, joined with in-house developments, make feasible in the soft X-ray regime, experiments accessing liquid layers and their interfaces with solids, opening up to completely new research fields with respect to traditional surface science, such as corrosion and battery research, with specifically designed electrochemical cells suitable for APXPS measurements [8].

#### The SPECIES & HIPPIE beamlines

SPECIES is a soft X-ray beamline on the 1.5 GeV ring. It covers a wide photon energy range of 30–1500 eV with variable polarization and high photon flux in the low photon energy range [9]. The optics provide a moderately focused beam (~100 µm). The beamline covers an energy resolution typical for soft X-ray beamlines of resolving power values of the order of 5000–10000 over nearly the entire photon energy range. The main instrument of the APXPS endstation is the Phoibos 150 NAP electron energy analyzer from SPECS GmbH, equipped with a delay line detector (DLD). The APXPS endstation is used to study solid–gas interfaces and has sample environments allowing for experiments in the fields of, among others, catalysis research, material characterization, and thin film deposition, utilizing dedicated cells.

HIPPIE, on the 3 GeV ring, covers a wider photon energy range than SPECIES (250 to 2500 eV), also with variable polarization [10]. It has two branches, each with its own permanently installed endstation dedicated to APXPS, that is, one for solid–gas experiments (SGE) and one primarily, but not limited to, for solid–liquid studies (SLE). The optics provide a spot size of 65 µm × 25 µm on the SGE and 60 µm × 50 µm on the SLE and a resolving power up to 35000. The SGE branch features a Scienta Omicron HiPP-3 analyzer with microchannel plate detector and camera up to 120 Hz frame rate (soon to be upgraded to a DLD). The SLE branch employs a SPECS Phoibos-NAP spectrometer equipped with a 2D DLD. Both the SGE endstation at HIPPIE and the APXPS endstation at SPECIES, use the so-called “cell in cell” method, whereby a small ambient pressure cell is inserted into the UHV chamber where it makes a seal-tight connection to the lenses of the electron analyzer, from which is separated via a small aperture cone [11]. This allows for the creation of the ambient pressure environment inside the cell while still maintaining UHV conditions on the outside, which enables the connection to the synchrotron beamline. The synchrotron radiation enters the cell through a thin membrane, which can hold the necessary pressure in the millibar range inside the cell. The endstations are also equipped with complementary instruments (e.g., mass spectrometers) mainly allowing researchers to probe gas phase products and connect this information easily to the XPS data acquired from the surface. Common in-vacuum surface preparation tools (e.g., Ar ion sputtering and sample annealing) are additionally available, which allows for preparing sample surfaces before commencing the APXPS experiments. Both beamlines have other external light sources (e.g., to provide solar radiation or UV light) available to all users in order to perform photocatalysis experiments. HIPPIE offers also a catalytic reactor to expose samples up to 1 bar gas atmosphere and 900 °C connected to the main UHV system. In addition, a polarization modulation infrared reflection absorption spectroscope (PM-IRRAS) is also available, enabling simultaneous APXPS and IRRAS measurements.

**Figure 1:**
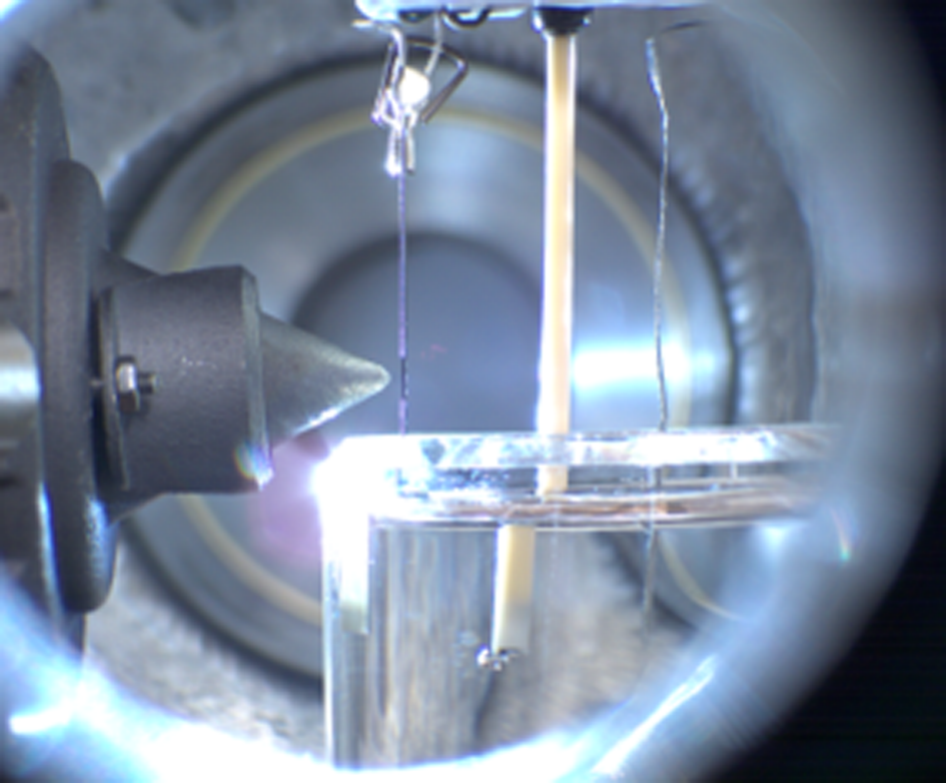
Picture of the electrochemical APXPS setup at HIPPIE. The EEA on the left is facing the working electrode. The three-electrode cell is then further composed of reference and counter electrodes. The electrodes are immersed in a beaker with a liquid electrolyte. The system is at equilibrium at the vapor pressure of the liquid. The picture was taken by Robert Temperton. This content is not subject to CC BY 4.0.

The SLE at HIPPIE is designed as a large back-filled vacuum chamber with two manipulators and base pressure in the 10^−6^ mbar range. Access to the chamber is realized via a large front door. This offers a versatile and flexible system where various custom setups and sample environments can be installed. The primary setup used at the SLE is an electrochemical (EC) cell; a liquid microjet is also used to a smaller extent. In the EC setup, a three-electrode setup is immersed and retracted from a beaker of liquid electrolyte, forming a thin, electrochemically active meniscus on the working electrode that can be probed using APXPS or XAS, see Figure 1. Electrochemistry is controlled via a Biologic SP200 potentiostat.

### Catalysis

Heterogeneous catalysis is one of the most prominent and long-standing applications of APXPS and one of the primary drivers of its development [4–5,12]. APXPS extends the tradition of UHV-XPS surface science by enabling direct, operando observation of catalytic surfaces under reaction conditions. This allows for full life cycle characterization of catalysts, offering insights into surface structure, composition, and dynamic behavior during catalysis. Thanks to its intrinsic surface sensitivity, APXPS is uniquely positioned to probe the active sites on catalytic materials. These surface sites, often structurally undercoordinated and electronically distinct from the bulk material, drive the transformation of reactants into products. While catalysts regenerate at the end of each cycle, their surfaces can be highly sensitive and unstable under ambient conditions, necessitating time- and environment-resolved measurements to capture their true reactive state.

Particularly important is the combination of APXPS with complementary techniques that could simultaneously explore the sample. At HIPPIE, PM-IRRAS can be performed in situ and operando. With IRRAS, vibrational information is available, which greatly complements the electronic information obtained by photoelectron spectroscopy. This combination enables a more complete picture of surface reaction mechanisms. The examples discussed here focus on “model catalysts”, that is, systems where the active phase is well defined and often prepared on single crystals. While studies on industrial catalysts are limited by charging effects (insulating oxide-supported samples) and higher pressure requirements, model systems remain invaluable for fundamental insights.

We now turn to specific case studies from MAX IV’s APXPS beamlines. We will not concentrate on the typical reactions (e.g., CO oxidation, ethylene epoxidation, and methanol oxidation) already deeply reviewed elsewhere [5,12–13]. We will instead discuss a few catalysis niche cases dealing with complex catalytic reactions and instrumentation development which highlight better the use of advanced light sources in probing the structure and dynamics of nanostructured materials and instrumental developments that enable novel investigations of materials under operando conditions. We will therefore discuss scientific examples about single-atom catalysts and progressing to confined catalysis, time-resolved measurements, photocatalysis, and atomic layer deposition.

#### Single-atom catalysts

Single-atom catalysts (SACs) have emerged as a frontier in (electro)catalysis, combining exceptional catalytic activity with optimal utilization of precious metals such as Rh, Pd, and Pt [14–15]. Their atom-level dispersion maximizes surface exposure and minimizes metal consumption, which is critical for both economic and environmental sustainability. However, their inherently low site density and metastability under reaction conditions present significant challenges for both synthesis and in operando characterization. In particular, photon-hungry techniques like APXPS require the high brilliance provided by fourth-generation synchrotron sources to probe such dilute systems.

From a synthetic perspective, stabilizing isolated metal atoms against agglomeration, especially at elevated temperatures, requires advanced strategies. Accordingly, diverse approaches have been developed to realize stable SACs across a wide range of metals and supports. Among these, site-selective atomic layer deposition [16], supported catalytically active liquid metal solutions (SCALMS) [17–18], and cluster encapsulation via strong metal–support interaction (SMSI) [19] have shown promise. While these methods have been successfully characterized using APXPS, the systems themselves were not investigated under catalytic turnover conditions and are therefore not discussed further here.

Instead, we focus on a study by Vesselli and co-workers that directly addresses catalytic activity in a biomimetic SAC system [20–21]. In their work, a cobalt single-atom biomimetic model catalyst is based on a self-assembled monolayer of Co-porphyrins grown on an almost free-standing graphene sheet. This well-defined platform mimics the structure of metal–nitrogen–carbon catalysts used in oxygen reduction reactions (ORRs).

**Figure 2:**
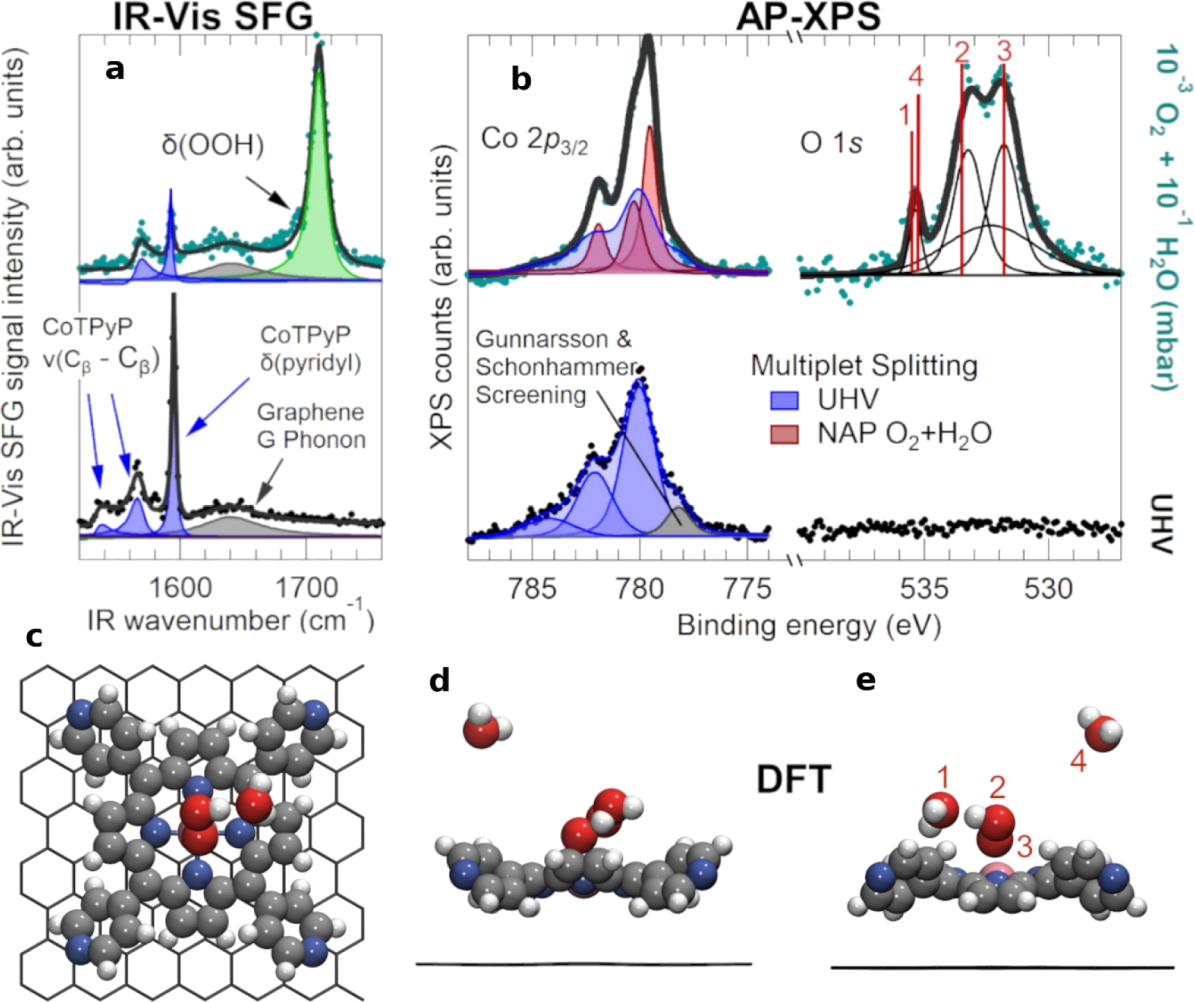
Spectroscopic and geometric characterization of the hydroperoxyl–water complex. (a) IR–vis SFG spectra of the pristine CoTPyP/GR layer in UHV (bottom) and in 10^−3^ mbar O_2_ +10^−1^ mbar H_2_O at room temperature (top); experimental data (markers) are reported together with the best fit (continuous lines) and deconvolution envelopes (filled profiles). (b) NAP-XPS spectra of the Co 2p_3/2_ (left, *h*ν = 910 eV) and O 1s (right, *h*ν = 660 eV) core levels corresponding to the same conditions as in (a); red vertical lines indicate the binding energies obtained for the optimized O_2_H–H_2_O complex on CoTPyP/GR from ab initio DFT calculations for different O 1s core levels, as labeled in (e): 1 − physisorbed/ wetting water, 2 − OOH, 3 − OOH, 4 − gas phase water. (c) Top view of the DFT-optimized model of the O_2_H–H_2_O/Co/TPyP/GR system. (d,e) Side views of the same system with an additional water molecule in the gas phase from two different points of view, to show the order-2 rotational symmetry of the CoTPyP, with a saddle shape macrocycle and peripheral pyridyl groups alternatively rotated by +39° and −39° with respect to GR. Figure 2 was reproduced from [21] (© 2022 F. Armillotta, et al., published by American Chemical Society, distributed under the terms of the Creative Commons Attribution 4.0 International License, https://creativecommons.org/licenses/by/4.0).

Using a combination of results obtained from APXPS at HIPPIE and pump–probe infrared–visible sum-frequency generation (SFG) in a dedicated setup at the Department of Physics of the University of Trieste [22], and supported by DFT, the authors demonstrated that the Co-porphyrin SAC stabilizes a hydroperoxyl–water (O_2_H-H_2_O) cluster in O_2_+H_2_O atmosphere at room temperature [21]. This is considered the fundamental reaction intermediate in the ORR. In particular, the configuration of the O_2_H radical could drive the selectivity for 2e^−^ vs 4e^−^ pathway ORR. The APXPS measurements revealed distinct changes in the Co 2p and O 1s core levels consistent with the formation of this reactive intermediate. Figure 2 shows spectroscopic evidence for the O_2_H–H_2_O/Co complex, highlighting the power of operando APXPS to capture chemically relevant intermediates on SACs under realistic reaction environments. This example highlights the unique ability of APXPS, particularly when combined with complementary vibrational spectroscopies, to provide molecular-level insight into active sites and mechanisms in single-atom catalysis.

#### 2D confined catalysis

Confined catalysis is an emerging field that investigates how spatial constraints can influence the efficiency, selectivity, and performance of catalytic reactions. This approach leverages the principles of confinement within various nanostructures (pores, cavities, or interlayer regions of materials) to create unique reaction environments that can significantly alter the behavior of catalytic processes. The confined environment can affect how reactants approach each other, stabilize reaction intermediates, and influence transition states, often lowering activation energies and altering reaction pathways [23–25].

**Figure 3:**
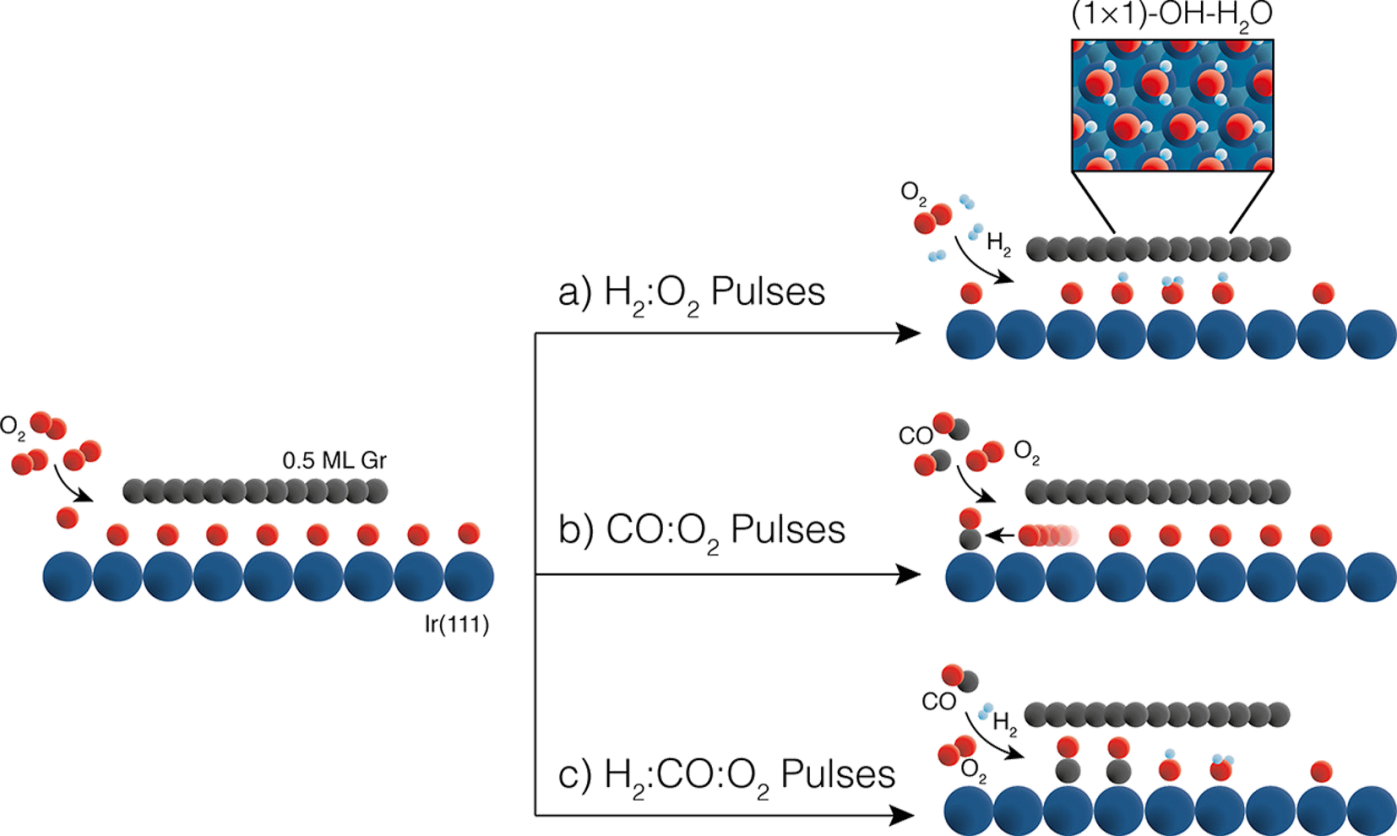
Schematic representation of the three undercover reactions studied. Starting from oxygen-intercalated Gr/Ir(111) (left), we expose the system to 50 s pulses of (a) a hydrogen-rich mixture resulting in the formation of OH−H_2_O under graphene (inset: ball model of the (1 × 1)- OH−H_2_O undercover structure), (b) a CO-rich mixture resulting in the removal of intercalated oxygen, and (c) a combined H_2_:CO-rich mixture resulting in the hydrogen-assisted intercalation of CO. Figure 3 was reproduced from [28] (© 2022 V. Boix et al., published by American Chemical Society, distributed under the terms of the Creative Commons Attribution 4.0 International License, https://creativecommons.org/licenses/by/4.0).

A particularly interesting case is “undercover catalysis”, where the void space between a 2D material and a catalytic surface is exploited. Materials such as graphene [26–28], hexagonal boron nitride (hBN) [29], and transition metal dichalcogenides [30] are widely studied for this purpose. Boix, Knudsen and collaborators combined APXPS with gas pulsing with varied composition to repeatedly form and remove undercover reaction products. Specifically, they studied CO and H_2_ oxidation below oxygen-intercalated graphene flakes partially covering an Ir(111) surface, as illustrated in Figure 3 [28].

**Figure 4:**
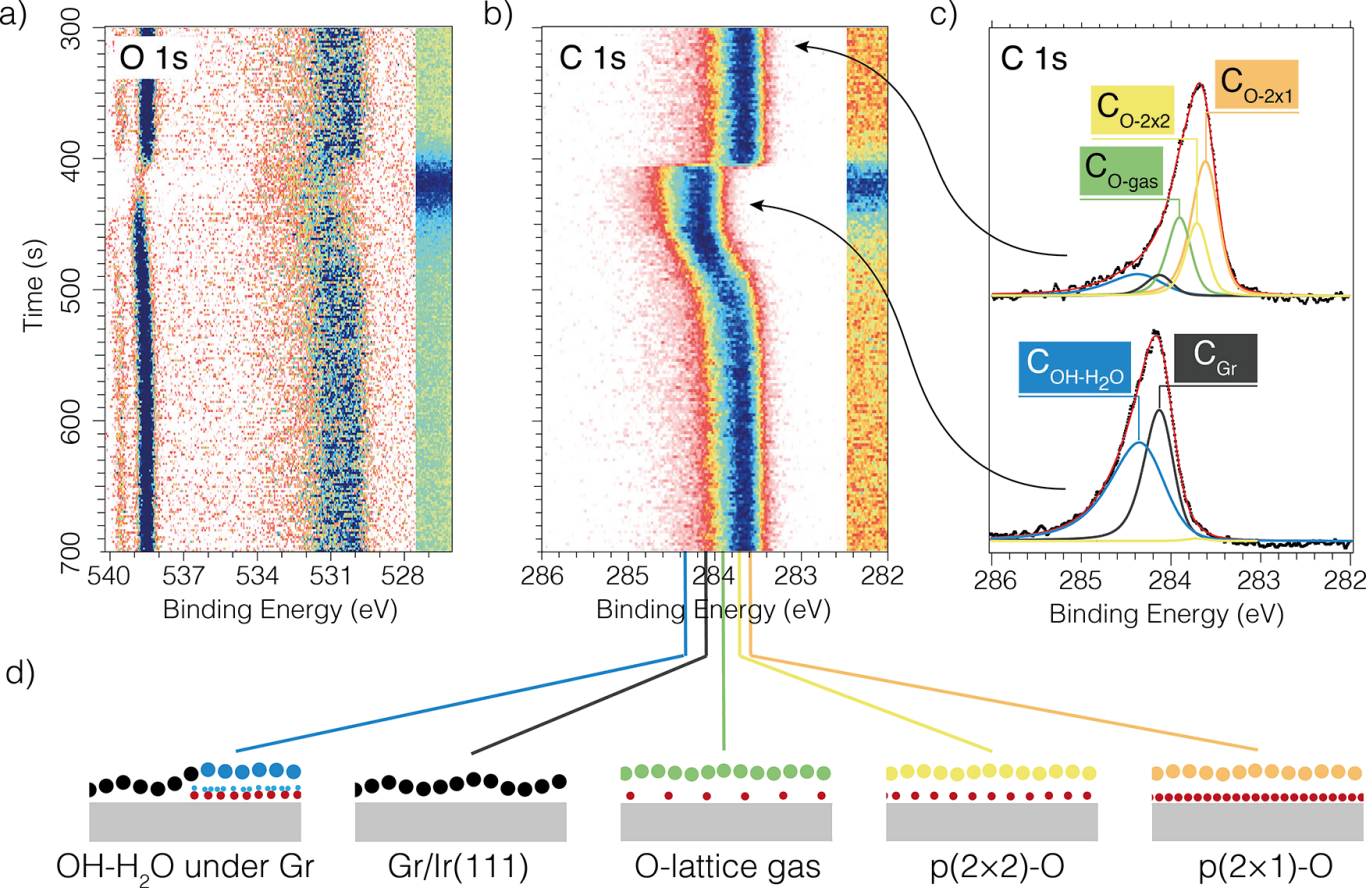
Hydrogen oxidation reaction under ~0.5 ML Gr on Ir(111) at 370 K and 1 mbar total pressure. (a, b) 2D image plots of (a) O 1s and (b) C 1s during one hydrogen-rich pulse (50 s of a hydrogen-rich mixture H_2_/O_2_ 9:1 sccm). (c) Average spectrum of 10 individual C 1s spectra recorded before and during the pulse. (d) Schematic representation of the five Gr/Ir(111) intercalation structures that define the five components used to fit the data. The position of each C 1s component is indicated with a line to the binding energy axis in panel (b). See main text for exact values. Figure 4 was reproduced from [28] (© 2022 V. Boix et al., published by American Chemical Society, distributed under the terms of the Creative Commons Attribution 4.0 International License, https://creativecommons.org/licenses/by/4.0).

Although the reaction products (H_2_O and CO_2_) were below the APXPS detection limit, the C 1s signal proved sensitive enough to monitor the dynamics of undercover catalysis during gas pulsing (see Figure 4). Their results revealed that H_2_ promotes the formation of a dense OH–H_2_O phase below the graphene. In contrast, CO alone showed minimal intercalation and instead scavenged oxygen. When CO and H_2_ were pulsed together, hydrogen modified the undercover chemistry: the formation of the OH-H_2_O phase lifts the graphene flake and allows the CO to intercalate and then oxidize.

**Figure 5:**
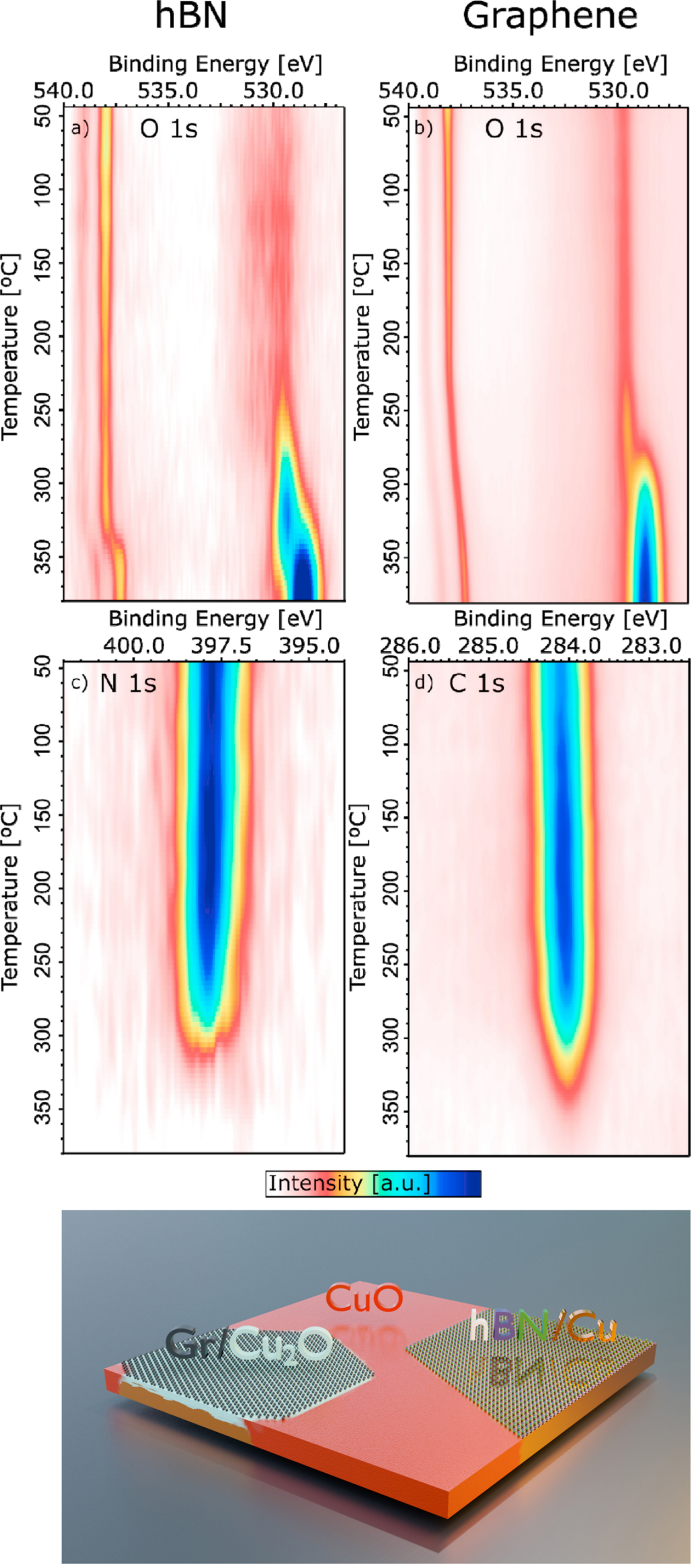
(Top) hBN and graphene protected Cu foils. (a, b) O 1s and (c, d) N 1s and C 1s 2D intensity plots as a function of temperature for hBN/Cu (a, c) and graphene/Cu (b, d). The spectra are recorded in 2 mbar O_2_, with a photon energy of 1070 eV. The intensity is normalized to the background at lower binding energy with respect to the peak. The fingerprint of Cu_2_O is the O 1s peak at about 530 eV, while CuO is at 529 eV. (Bottom) Sketch of the surface at 250 °C. Figure 5 was reproduced from [31] (© 2021 M. Scardamaglia et al., published by American Chemical Society, distributed under the terms of the Creative Commons Attribution 4.0 International License, https://creativecommons.org/licenses/by/4.0).

Rather than studying the reaction below 2D materials, Scardamaglia et al. investigated graphene and hBN as protective layers for copper metal surfaces in a reactive environment [31]. In operando experiments with a linear temperature ramp in 2 mbar O_2_, bare copper rapidly oxidized to Cu_2_O at room temperature and further to CuO at ~200 °C. However, with hBN or graphene layers, oxidation was significantly delayed. Both 2D materials retard the oxidation of Cu by more than 120 °C, but with different kinetics. The behavior of hBN/Cu is relatively simple: the hot copper surface is well shielded from oxygen, and even if some oxygen penetrates through wrinkles or boundary areas, the insulating nature of hBN prevents rapid copper oxidation. This protection remains effective until the coating layer is fully and quickly etched away (up to 300 °C). Beyond this point, the exposed hot copper surface undergoes rapid oxidation, transforming from metallic Cu to Cu_2_O, and, eventually, to CuO within a small temperature range. In contrast, with graphene/Cu, although the protective layer degrades at a slightly higher temperature than hBN, oxygen begins to intercalate beneath graphene at around 220 °C, initiating a slower, undercover oxidation of copper to Cu_2_O. This process is a galvanic reaction facilitated by graphene’s high conductivity, enabling electron transfer from copper to oxygen atoms. However, the graphene/Cu_2_O hybrid structure slows further oxidation to CuO due to limited oxygen availability, as the reaction occurs beneath the surface. This is significant because it offers a method to stabilize the Cu_2_O phase at high temperatures, preventing its conversion to CuO. As the temperature increases further, CuO formation and graphene etching occur simultaneously. This is summarized in Figure 5 where the 2D intensity plot vs temperature of O 1s and N 1s or C 1s, for hBN or graphene, respectively, are reported.

#### Time-resolved catalysis

Catalysts are inherently dynamic systems. The optimal catalyst should have a nanostructure that enhances activation kinetics, contain active sites that are dynamically stabilized, and be in a state of deviation from chemical equilibrium under reaction conditions. Therefore, fully describing a functional catalyst requires addressing a range of dynamics across multiple temporal scales. To examine such a dynamic at the atomic level, we must ensure a well-controlled environment that is relevant to catalytic conditions. This includes enhancing the temporal resolution of APXPS measurements. Ultimately, the goal is to understand the heterogeneity of a catalytic system with greater specificity regarding site, time, chemistry, and environment.

Recent years have seen growing interest in time-resolved APXPS studies, particularly those enabled by DLDs [32–37]. These detectors offer microsecond-scale time resolution, unlocking the ability to track fast chemical transformations. In this section, we focus on ultrafast measurements that are possible with the new DLDs at the HIPPIE and the SPECIES beamlines. These DLDs are built like a very tight check pattern of electrical wires. Every impinging electron sends an electrical signal along two orthogonal wires and its location is determined by the readout electronics based on the time of flight. This architecture allows for high-speed acquisition (only limited by the speed of the readout electronics), either as continuous time-resolved data or in pump–probe mode. In continuous mode, spectra are recorded in sequence without scanning the analyzer’s retardation voltage. To observe quick, periodic changes in the APXP spectra, we apply fast gas pulses to the catalyst surface [38–40]. During continuous acquisition, APXP spectra are measured one after the other with a high time resolution and for many gas pulses. This enables ultrafast capture but results in the measured signal being convoluted with the analyzer’s transmission function.

**Figure 6:**
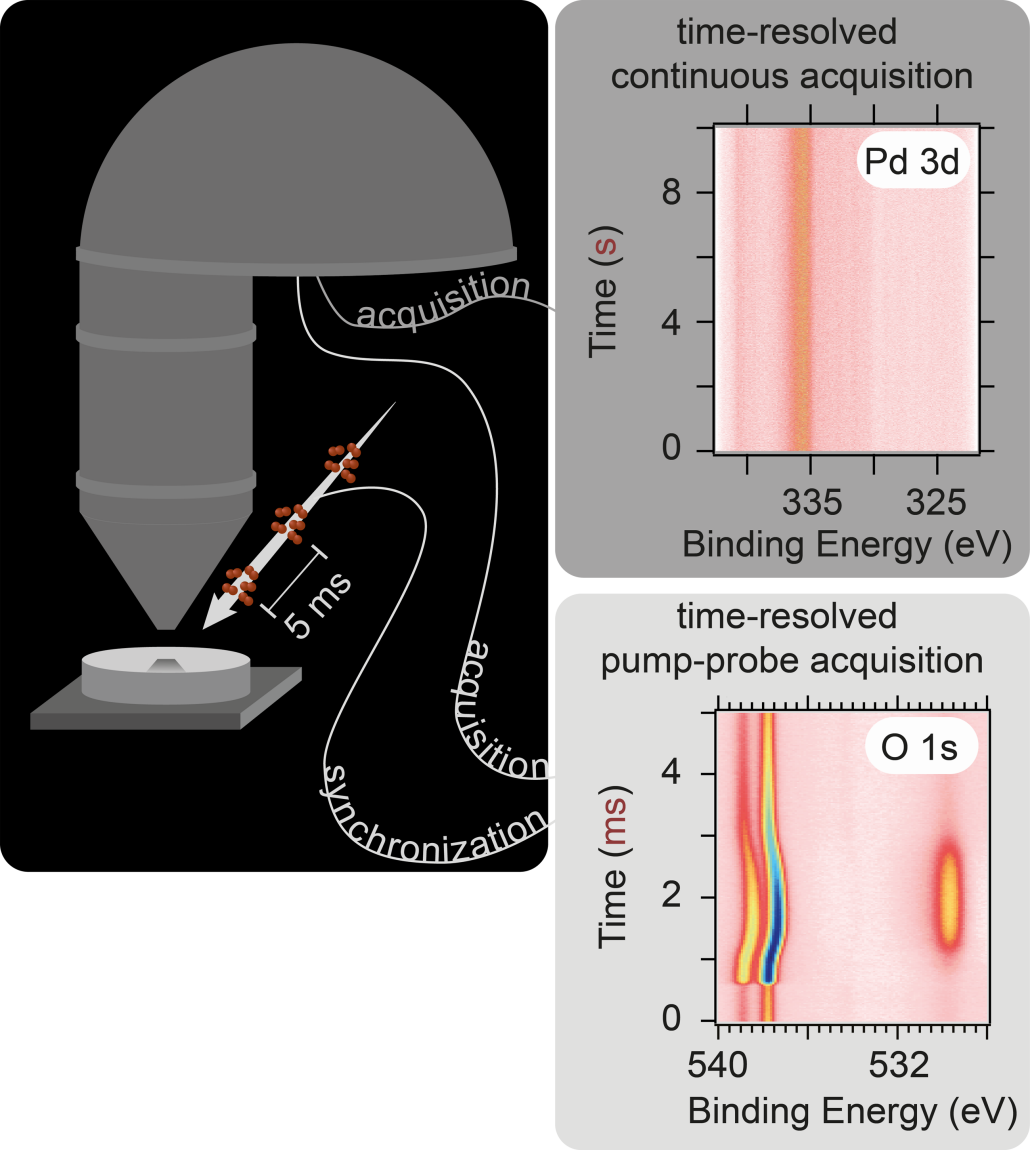
Sketch of the two different measurement mechanisms possible at the DLD at HIPPIE.

During pump–probe acquisition, a synchronization signal triggers spectral acquisition with each external perturbation (e.g., gas pulse). This makes it possible to assign a time difference between gas pulse and spectrum acquisition to every measurement. Thus, scanning of the retardation voltage becomes possible, that is, the analyzer transmission is accounted for. Since event averaging over many gas pulses is done in the analyzer, the measurement can be run very fast. A significant difference in the direct analysis can immediately be observed when comparing the data measured with the two acquisition techniques in Figure 6. The spectroscopic changes induced by the modulation can often be directly seen in pump–probe data, while post-processing is necessary for continuous measurements to obtain similar information. Examples of the two different measurement schemes are presented below. The data are taken from two experiments performed at the SLE of the HIPPIE beamline, while exposing a catalyst to gas pulses.

**Figure 7:**
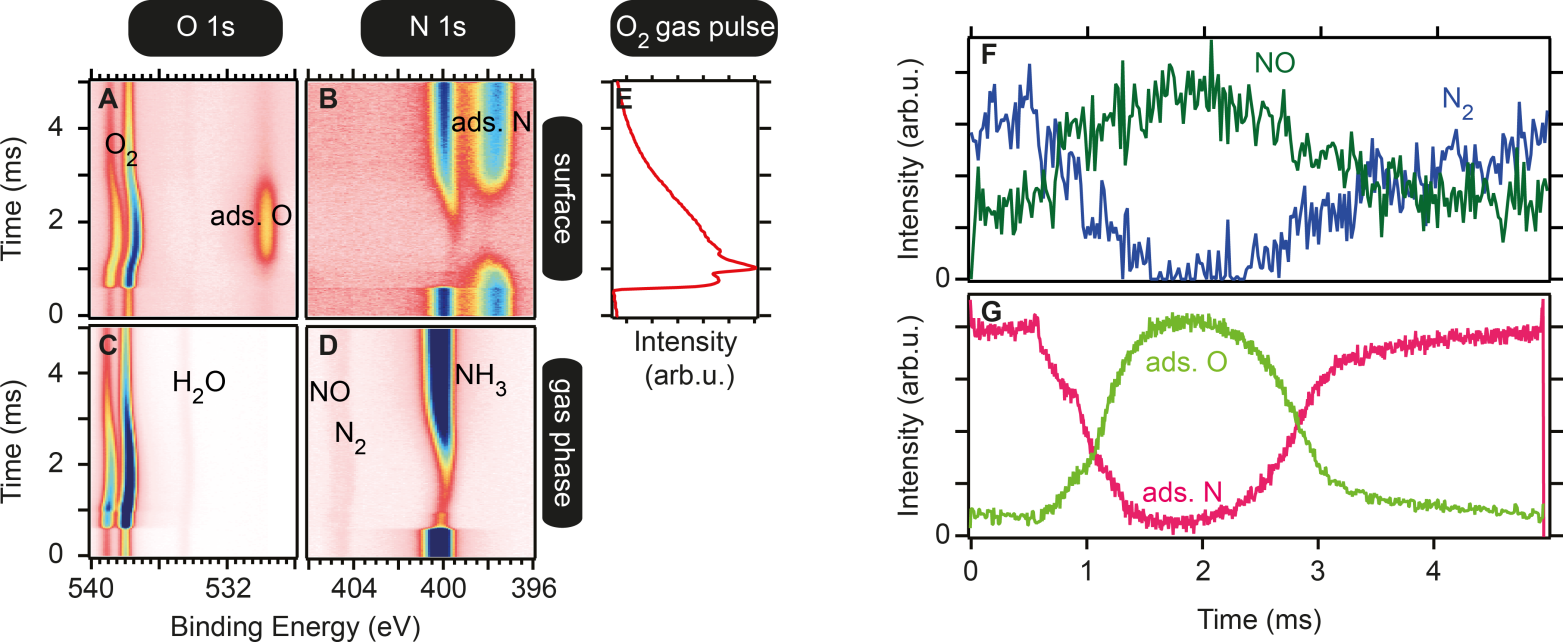
(A–D) O 1s and N 1s core level spectra in surface- and gas phase-sensitive positions measured in pump–probe mode during experiment 1. (E) Profile of the injected gas pulse over time. (F) Time evolution of the nitrogen-containing products of NH_3_ oxidation. (G) Time evolution of the surface adsorbates.

**Ammonia oxidation (microsecond time-resolution, pump–probe mode):** This experiment used 150 µs wide 170 V voltage pulses with a repetition rate of 5 Hz to control O_2_ gas injection (piezoelectric valve) in a background of NH_3_ (15 sccm) and O_2_ (10 sccm) achieving 3.8 mbar (the local pressure at the sample surface is much higher) total pressure at 460 °C on a PtRh(100) catalyst. Spectra were acquired at 200 kHz (5 µs time resolution), averaging over 720,000 pulses. One measured spectrum is sorted into the 2D image shown in Figure 7 by using the time difference between the pump signal and the probing signal. The 2D image averages over 720,000 gas pulses.

Using the O 1s and N 1s core levels (Figure 7A–D), it is possible to follow both the surface composition and the catalyst selectivity by observing the gas phase (with a slightly retracted sample). The time evolution of NO and N_2_ taken from curve fits of the N 1s gas phase spectra (Figure 7D) is shown in (Figure 7F) while the evolution of the dominant surface adsorbate taken from curve fits in the O 1s (Figure 7A) and N 1s (Figure 7B) spectra is shown in Panel (Figure 7G). The main conclusion here is that the selectivity of the catalyst towards NO formation increases, similar to the increase in coverage of the surface with oxygen. Likewise, an increasing coverage of adsorbed nitrogen leads to an increased selectivity towards N_2_.

Previously, Resta et al. [41] could correlate the production of NO with the formation of surface RhO_2_ for four different NH_3_-to-O_2_ ratios. In the presented example, studying the reaction in a time-resolved manner, it was possible to study a continuous range of different reactant ratios and their effect on the product composition within one experiment, which makes them inherently comparable. Thus, this pump–probe acquisition mode enables structure–activity correlations with high time resolution.

**Figure 8:**
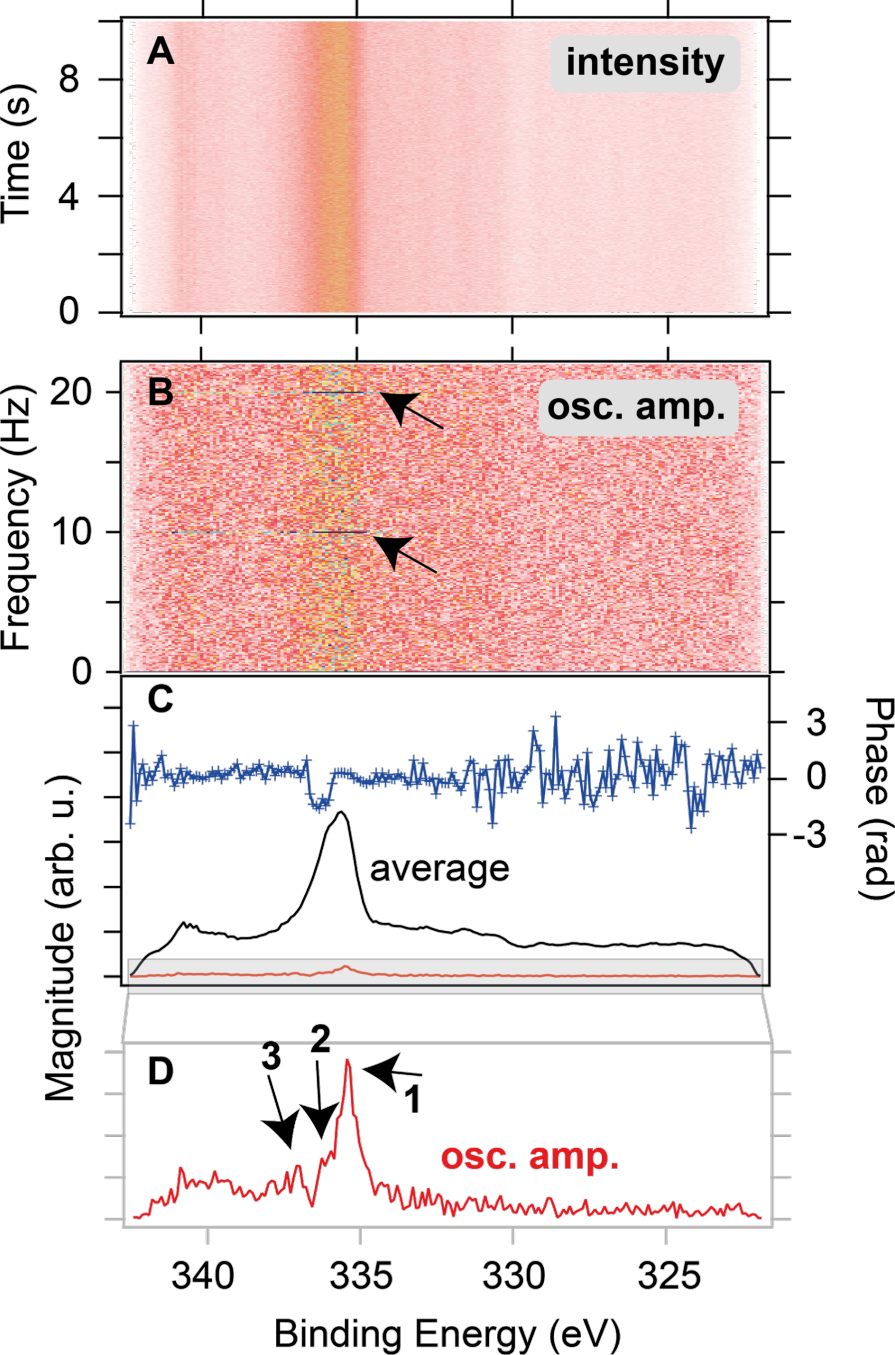
(A) Pd 3d core level spectra measured in continuous mode during experiment 2 in surface sensitive position. (B) Magnitude of the Fourier transform as a function of frequency and binding energy. (C) Oscillation amplitude and phase at the fundamental frequency (10 Hz), together with the time average of the spectra. (D) Magnified oscillation amplitude.

**Ethylene oxidation (millisecond time-resolution, continuous mode):** In a second experiment, 200 µs pulses of C_2_H_4_ were introduced into a 10 sccm O_2_ flow over a polycrystalline Pd catalyst at 365 °C with a repetition rate of 10 Hz and a total pressure of 3.7 × 10^−3^ mbar (the local pressure at the sample surface is much higher). The resulting APXP spectra shown in Figure 8 were measured continuously in time-resolved snapshot mode with an acquisition frequency of 1 kHz (1 ms time resolution). One measured spectrum covers approximately 100 pulses.

While the raw data showed no visible modulation (Figure 8A), Fourier analysis of the spectra as described in detail elsewhere [42–43] revealed an oscillatory response as highlighted by the black arrows in (Figure 8B) at the driving frequency of the gas pulses and higher harmonics. If the magnitude and phase of the Fourier transform at the fundamental frequency (i.e., 10 Hz) are extracted and plotted (Figure 8C), a clear footprint of oscillating components can be seen in the blue phase signal at 335–337 eV. Magnification of the oscillation magnitude even reveals three different oscillating components within the broad Pd 3d_5/2_ peak seen in the time average in (Figure 8C). Here, the phase signal shows that the middle component (2) oscillates in antiphase with the other two (1,3); when 2 increases, 1 and 3 decrease and vice versa. Thus, even though the oscillating signal picked up during the measurement is small in comparison to the time average of the spectra, Fourier analysis can reveal the periodic spectroscopic answer.

**Comparing continuous and pump–probe acquisition:** Pump–probe mode offers higher sensitivity to transient signals and smaller datasets but requires precise periodic modulation. It is ideal for fast, repeatable processes. Continuous acquisition, in contrast, is suitable for systems with self-sustained or aperiodic oscillations. Fourier analysis allows for selective extraction of oscillating signals, removing static background contributions. Both modes have proven indispensable in revealing the real-time dynamics of catalytic reactions, and they have only become possible with the recent advances in detector development. Their availability at HIPPIE and SPECIES is a major asset for APXPS studies.

#### Photocatalysis

Utilizing solar energy to drive chemical reactions is a central focus of sustainable energy research. Replacing fossil fuels with clean and renewable energy sources is one of the most important challenges for modern human civilization. Reactions such as water splitting, CO_2_ reduction, and pollutant degradation rely on photocatalysts that absorb light, generate electron–hole pairs, and drive redox reactions at interfaces. For the rational and methodical design of a more sophisticated photocatalyst, it is essential to combine the photocatalyst’s performance with mechanistic knowledge under operando reaction conditions.

**Figure 9:**
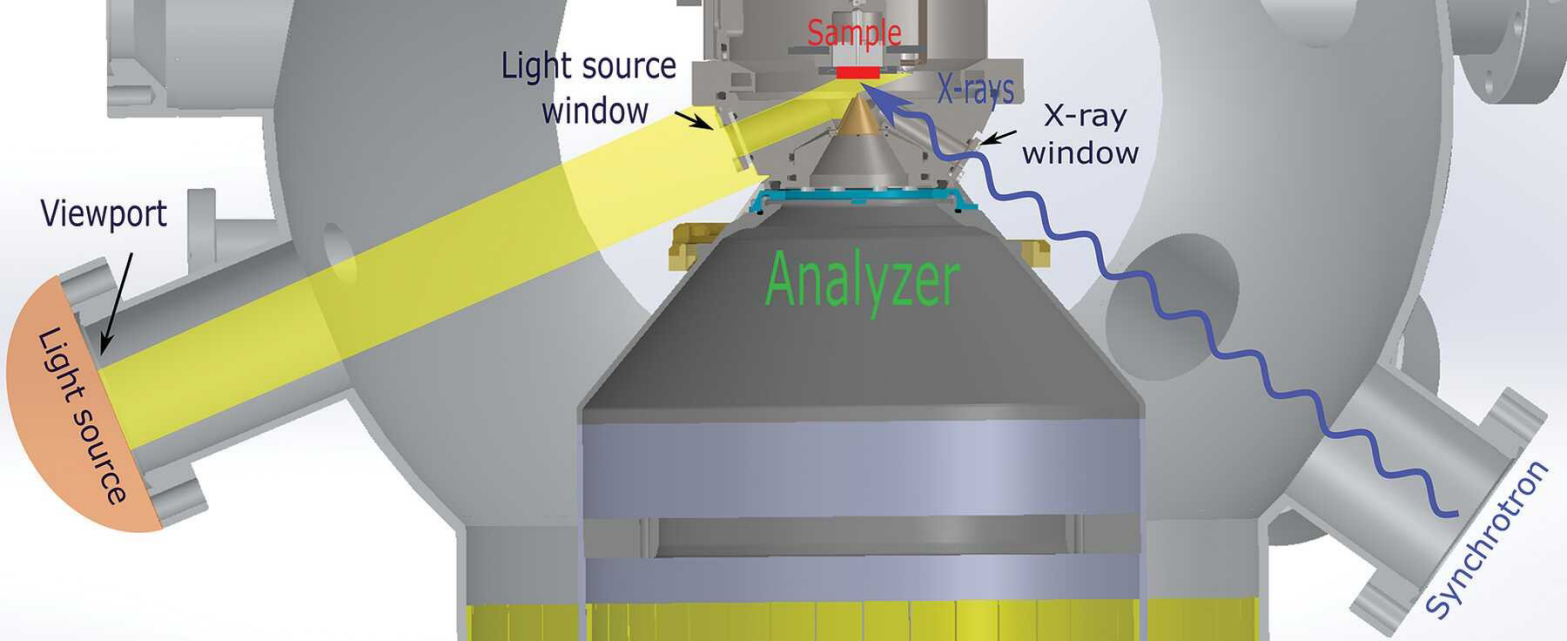
Schematic layout showing the alignment between external light sources, beamline, and analyzer nozzle in the analysis chamber with AP cell at the SPECIES beamline at MAX IV Laboratory. Figure 9 was reproduced from [44] (© 2023 A. Klyushin et al., published by the International Union of Crystallography distributed under the terms of the Creative Commons Attribution 4.0 International License, https://creativecommons.org/licenses/by/4.0).

APXPS is uniquely suited to investigate such systems. Its surface sensitivity enables the detection of charge transfer processes, reaction intermediates, and active sites under operando conditions. Additionally, the kinetics of charge transfer and the role of specific chemical species or components functioning as charge donors or acceptors during photocatalytic reactions can be clarified using APXPS. At MAX IV, external light sources (e.g., UV lamps or solar simulators) are coupled to the HIPPIE and SPECIES endstations to allow for photoirradiation in both UHV and ambient pressure environments (Figure 9) [44].

**Figure 10:**
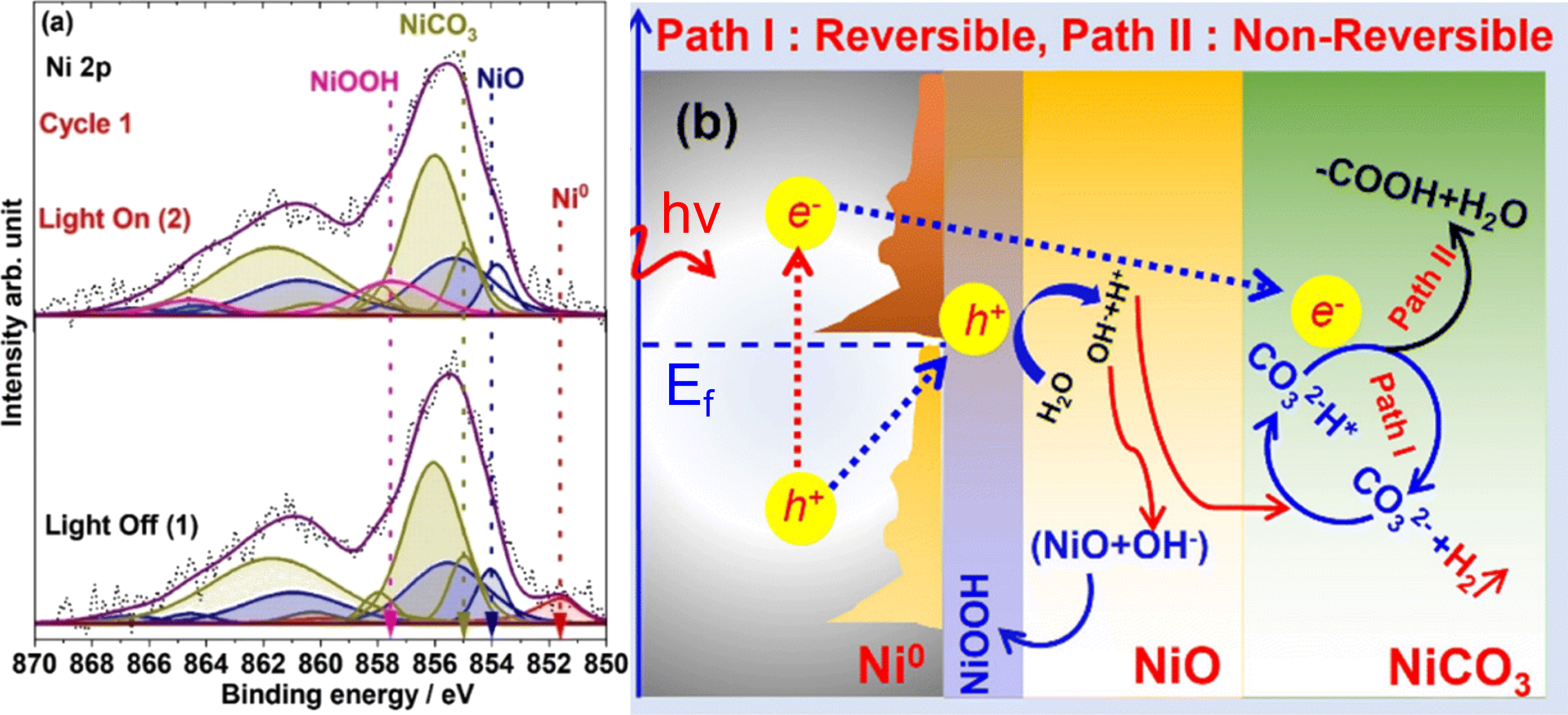
(a) Ni 2p XP spectra recorded at 1 mbar H_2_O pressure under dark (bottom spectra) and light illumination (top spectra) conditions. (b) Photocatalytic HER mechanism under light illumination 1 mbar H_2_O pressure. Figure 10 was reproduced from [45] (“Solar light driven atomic and electronic transformations in a plasmonic Ni@NiO/NiCO3 photocatalyst revealed by ambient pressure X-ray photoelectron spectroscopy“, © 2024 M. K. Ghosalya et al., published by The Royal Society of Chemistry, distributed under the terms of the Creative Commons Attribution 3.0 Unported Licence, https://creativecommons.org/licenses/by/3.0).

The most environmentally friendly alternative to fossil fuels is green hydrogen; however, producing it at a reasonable price remains challenging. A prominent application is photocatalytic water splitting for green hydrogen production. Urpelainen and colleagues clarified the mechanisms of photocatalytic hydrogen evolution reaction (HER) in a new prospective model system, the Ni@NiO/NiCO_3_ photocatalyst, under dark and illuminated conditions at 1 mbar of H_2_O [45]. Figure 10a illustrates how the complex Ni 2p spectrum is caused by multiplet splitting, shake-up, and plasmon loss structures. When the catalyst surface is dark, the Ni 2p_3/2_ structure can be well matched with three main components assigned to Ni^0^, NiO, and NiCO_3_. Upon illumination, the Ni^0^ peak vanishes, and the main Ni 2p peak broadens due to the emergence of an additional peak at 857.4 eV attributed to NiOOH. This indicates that photoexcitation triggers oxidation and structural changes, consistent with plasmon-driven electron excitation at the Ni/NiO interface. The authors propose a mechanism where photoexcited holes migrate to NiO and are trapped, facilitating water oxidation via an intermediate NiOOH species (Figure 10b).

APXPS thereby provides mechanistic insight into photoinduced surface chemistry, enabling rational catalyst design. Similar approaches can be extended to CO_2_ reduction, pollutant remediation, and other light-driven processes.

### Atomic layer deposition

Atomic layer deposition (ALD) is a critical thin-film technology widely used in semiconductors, nanoelectronics, energy storage, catalysis, and advanced coatings. It enables atomically precise control over film thickness and composition, even on topologically complex surfaces, making it essential for the fabrication of next-generation devices [46–48]. In the semiconductor industry, ALD enables the creation of nanoscale transistors and high-*k* dielectric materials, essential for shrinking transistor dimensions and enhancing performance in integrated circuits. Its precision, scalability, and ability to produce conformal films on intricate surfaces make ALD a cornerstone in the pursuit of miniaturization and enhanced performance in technology.

ALD achieves such high levels of growth control by a well-separated alternating exposure of a surface to precursors and co-reactants. Each reaction with the surface should be saturated and self-limiting, thus allowing the deposition of one monolayer to progress in a highly conformal layer-by-layer way, even on nanostructured materials of high aspect ratios. Traditional insights into ALD surface chemistry have come from ex situ or in vacuo XPS, often complemented by other surface characterization tools, used to characterize the resulting film [49–51], as well as to investigate chemical mechanisms cycle-to-cycle [51–52]; the latter procedure is often termed in situ XPS. However, such approaches are blind to the time-dependent dynamics that govern film nucleation and early growth. Operando APXPS overcomes this limitation, enabling direct observation of surface reactions cycle-by-cycle in real time [53].

ALD typically operates in the 10^−3^ to 1 mbar pressure range, ideally matched to the working range of APXPS. Time-resolved APXPS has proven especially powerful for studying the initial ALD cycles, where the chemical interactions at the surface are most critical [54–60]. At MAX IV, the SPECIES beamline has led the development of ALD studies using APXPS. A dedicated reaction cell was designed to replicate commercial ALD conditions, including independent pulsing of precursor gases, laminar flow across the sample, and substrate heating. The setup allows for independent control of precursor and co-reactant exposure and supports a variety of chemistries [60].

ALD is a key technology for the future development of smaller and more power-efficient devices. One step in this development is the replacement of silicon, which cannot reach the development goals set by international technological roadmaps [61]. A potential replacement for silicon oxide is a high-*k* oxide material such as HfO_2_, which allows for the creation of much smaller gate sizes. Another suggested change is to substitute the silicon channels with a III–V semiconductor material such as InAs, which, among other properties, has a narrower band gap [62]. However, one challenge to overcome with the InAs/HfO_2_ material is to obtain a clean interface between the materials, which is critical for the proper function of the potential devices. In situ and operando APXPS has been utilized in many works to study the deposition of HfO_2_ on InAs to elucidate the chemical reactions that occur as those layers are grown in order to better understand how to create sharp interfaces between the materials [55,57–58,63].

**Figure 11:**
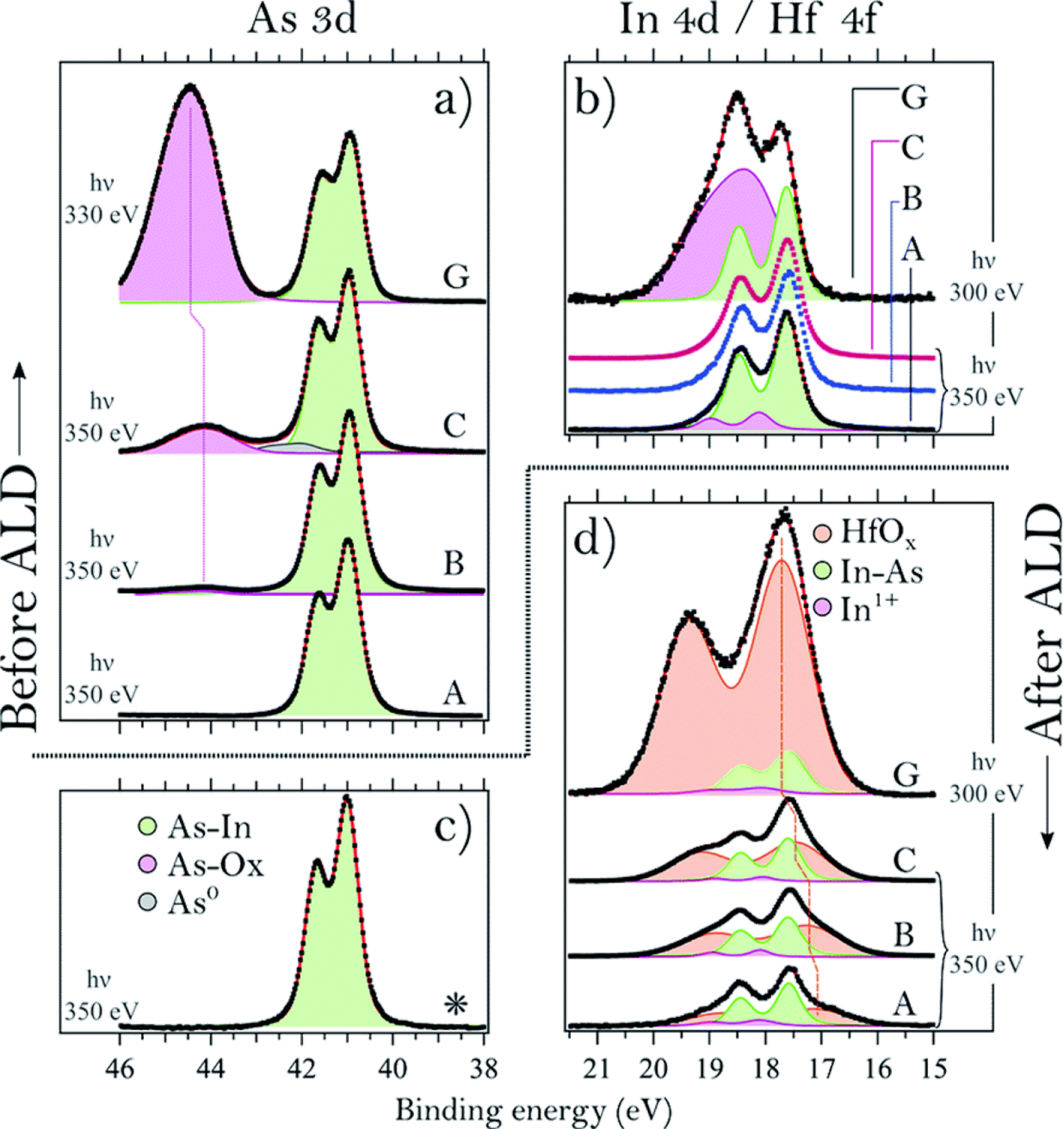
Sample A was initially cleaned by bombarding it with elemental hydrogen, thus removing all of the oxide components. Samples B and C were characterized to have 0.05 L, and 6.0 L of O_2_ exposure, respectively; sample G was treated with a water bath and is referred to as having a native oxide. (a) As 3d core level spectra of four different InAs samples with varying As oxide thickness measured before TDMAHf deposition. (b) In 3d spectra of the same samples showing the In oxide. (c) As 3d spectrum of sample C after the first TDMAHf half-cycle. (d) In 4d and Hf 4f spectra (which overlap in energy) acquired after the first TDMAHf half-cycle. Figure 11 was reproduced from [63] (“Oxygen relocation during HfO_2_ ALD on InAs “, © 2022 G. D'Acunto et al., published by The Royal Society of Chemistry, distributed under the terms of the Creative Commons Attribution 3.0 Unported Licence, https://creativecommons.org/licenses/by/3.0).

In one study by D’Acunto et al., the surface composition and thickness of the initial growth of HfO_2_ on InAs was investigated using tetrakis(dimethylamido)hafnium(IV) (TDMAHf) as the hafnium precursor [63]. Four InAs samples with varying native (samples A, B, and C) or thermal oxide (sample G) layers were exposed to TDMAHf at ~200 °C under ~10^−3^ mbar pressure. Results showed that TDMAHf interacts with the surface oxide to produce HfO_2_ already in the first half-cycle, a “self-cleaning” effect that removes native oxides from InAs and transfers oxygen to Hf. The resulting interface contained an In–O–Hf layer limited to approximately one monolayer. The second half-cycle is often done using water as the co-reactant and is the principal source of oxygen for the creation of HfO_2_ in the later ALD cycles. Figure 11 shows high-resolution photoelectron spectra of As 3d, In 4d, and Hf 4f core levels before and after the first TDMAHf half-cycle on the four samples. The oxide peaks of As and In disappear for all the thermal oxide (samples A–C), and HfO*_x_* peaks emerge consistently across samples with differing initial oxide thicknesses. The only exception was the native oxide sample (G); it showed stronger Hf 4f intensity, but still the formed HfO*_x_* thickness was estimated to be limited to one monolayer of approximately 5.6 Å.

**Figure 12:**
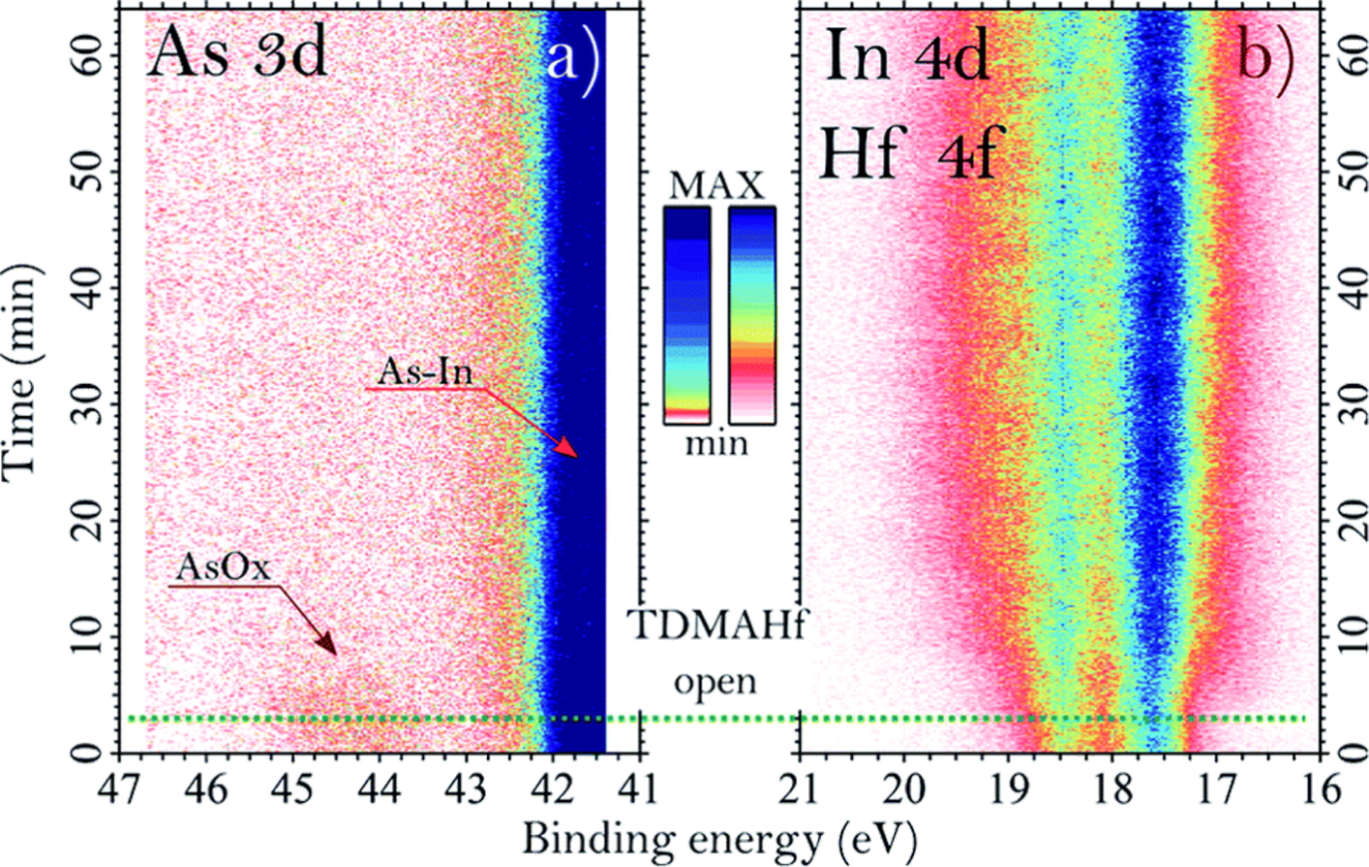
Time-resolved XP spectra acquired during the TDMAHf deposition on InAs showing (a) the As 3d core level and (b) the combined In 4d and Hf 4f region. The green dotted line indicates the time when the TDMAHf valve was opened. Figure 12 was reproduced from [63] (“Oxygen relocation during HfO_2_ ALD on InAs “, © 2022 G. D'Acunto et al., published by The Royal Society of Chemistry, distributed under the terms of the Creative Commons Attribution 3.0 Unported Licence, https://creativecommons.org/licenses/by/3.0).

Time-resolved APXPS (Figure 12) confirmed that the removal of surface oxides and the formation of HfO*_x_* occur simultaneously during precursor exposure. These results suggest that HfO_2_ can form even without an external oxygen source during the first half-cycle, using the available oxygen content on the surface, challenging traditional ligand-exchange descriptions of ALD chemistry.

### Electrochemical APXPS

Another field where APXPS has opened entirely new experimental possibilities is the study of electrochemical interfaces. Traditional XPS, limited to ultrahigh vacuum environments, has historically been unable to access solid–liquid interfaces, crucial for understanding electrochemical reactions in batteries, fuel cells, corrosion, and electrocatalysis. With the advent of ambient pressure setups and specially designed sample environments, APXPS now allows for direct probing of the chemical and electronic structure at solid–liquid interfaces under realistic conditions. This capability provides unique insight into surface reactions, charge transfer, and interface stability, which are central to the performance and degradation mechanisms of electrochemical systems. Electrochemical interfaces can be studied by APXPS using two primary cell types, namely, membrane-based flow cells and “dip-and-pull” systems. While flow cells are ideal for large currents and gas evolution, dip-and-pull cells, whilst more compromised from an electrochemical cell perspective, are more flexible and compatible with well-defined materials such as single crystals or layered structures [64]. Here, we outline some case-studies that use the dip-and-pull method available at HIPPIE.

The measurements can be performed in situ or operando. In the first, easier, case, the electrochemical reaction is performed with the sample immersed in the electrolyte, then all potentials are dropped, and the sample is pulled for APXPS measurements at the vapor pressure of the electrolyte. This method is used mostly for corrosion studies. In the second case, the potential is held during the XPS analysis, and a thin electrolyte film (meniscus) is maintained at the interface of the working electrode (WE) during measurement. This meniscus must be thin enough to allow photoelectrons to escape, yet thick enough to maintain electrical contact. The meniscus thickness depends on factors such as the wetting properties of the WE, liquid viscosity, surface tension, and pulling speed. This geometry enables true operando studies of solid–liquid interfaces and is particularly relevant for battery research.

#### Corrosion

Nanometer-thin and spontaneously formed oxide layers, also known as passive films, play a crucial role in the corrosion resistance of many advanced alloys. The current knowledge of the passive films’ structure and composition is largely derived from ex situ surface analysis using XPS and scanning tunneling microscopy. However, the findings of UHV-XPS do not accurately represent the genuine passive film/electrolyte interface. APXPS is essential to observe the onset and progression of corrosion and to gain a fundamental understanding of corrosion mechanisms.

**Figure 13:**
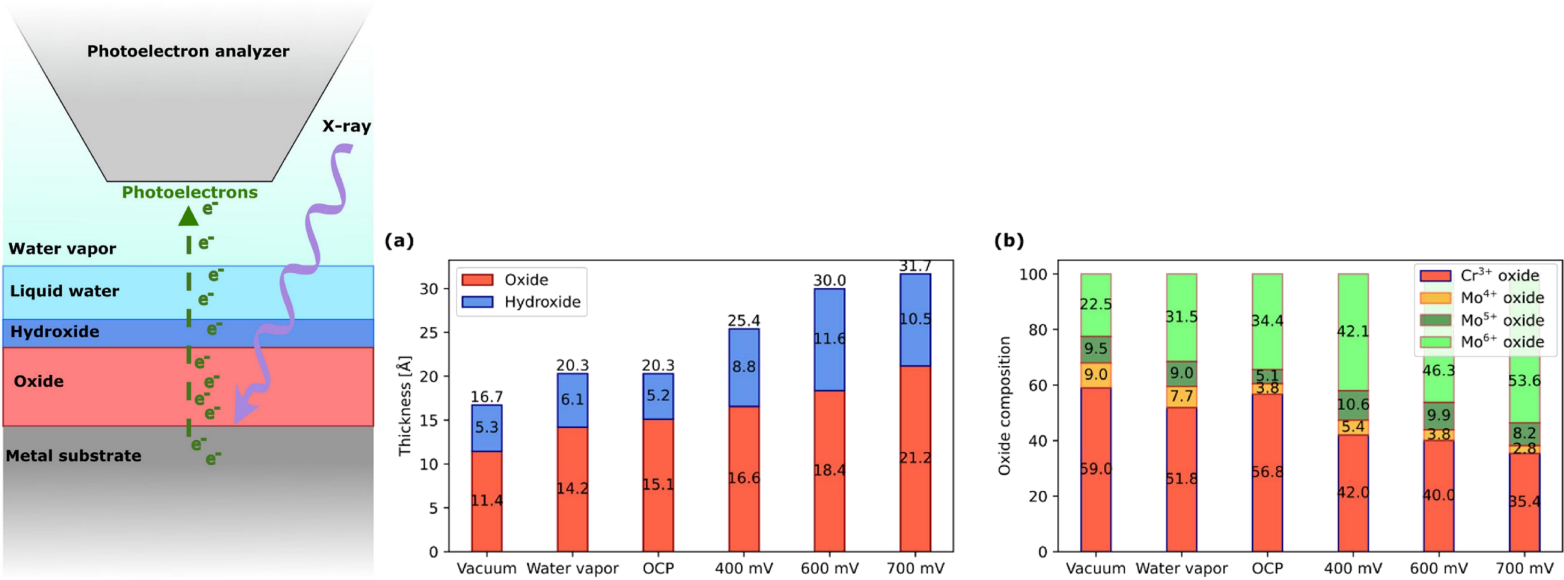
(Left) Layered model of surface region illustrating the metal substrate, oxide layer, hydroxide layer, liquid water layer, and the surrounding water vapor. Quantitative analysis. (a) Oxide and hydroxide thickness for different exposure conditions. (b) Total oxide composition for different exposure conditions. All potentials are reported against Ag/AgCl reference electrode. Figure 13 was reproduced from [66] (© 2023 A. Larsson et al., published by Elsevier, distributed under the terms of the Creative Commons Attribution 4.0 International License, https://creativecommons.org/licenses/by/4.0).

Lundgren’s and Pan’s groups studied in situ, in 17 mbar of water vapor, the electrochemical oxide growth and breakdown on Ni-Cr-Mo industrial alloys in a NaCl solution at different pH values, by following the anodic growth of the oxide film at potentials up to 700 mV vs Ag/AgCl [65–66]. The oxide, natively rich in Cr^3+^, undergoes a dynamic change and becomes enriched in Mo^6+^, with a chemical environment similar to that of pure MoO_3_. The accumulation of Mo in the passivated oxide film is believed to result from the depletion of Cr and the concurrent enrichment of Mo just beneath the native oxide layer, as reported in Figure 13.

**Figure 14:**
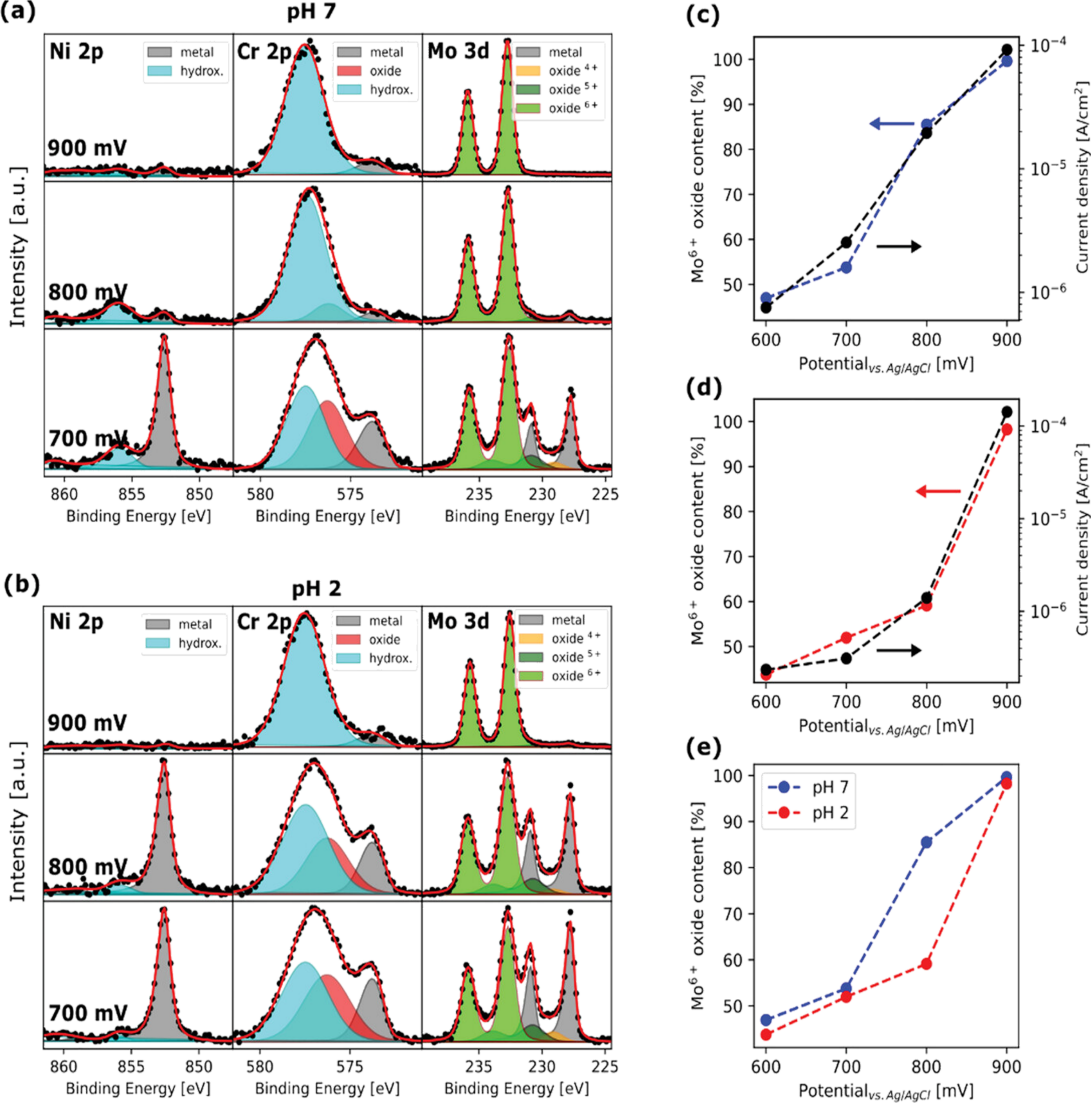
Passive film breakdown. (a) In situ APXPS spectra of Ni 2p_3/2_, Cr 2p_3/2_, and Mo 3d measured at pH 7 for the potential range of 700–900 mV. (b) In situ APXPS spectra of Ni 2p_3/2_, Cr 2p_3/2_, and Mo 3d measured at pH 2 for the potential range of 700–900 mV. (c) Mo^6+^ oxide content and current density after 10 min vs potential at pH 7. (d) Mo^6+^ oxide content and current density after 10 min vs potential at pH 2. Arrows indicate the axis corresponding to the data. (e) Comparison of Mo^6+^ oxide content at pH 7 and pH 2 vs potential. Figure 14 was reproduced from [65] (© 2023 A. Larsson et al., published by Wiley, distributed under the terms of the Creative Commons Attribution 4.0 International License, https://creativecommons.org/licenses/by/4.0).

The Ni-Cr-Mo alloy exhibited activity towards the oxygen evolution reaction (OER), which is responsible for initiating passivity breakdown due to the dissolution of Mo in its Mo^6+^ state. This mechanism contrasts with the typical transpassive breakdown behavior observed in Cr-containing alloys, where Cr^6+^ dissolution occurs at high anodic potentials, compromising the integrity of the passive layer. However, in this case, Cr^6+^ dissolution was not observed. Furthermore, at high current densities, the OER contributes to the localized acidification near the alloy’s surface, exacerbating metal dissolution. The passive film breakdown has been studied for different acidities of the electrolyte solution, as reported in Figure 14. A parallel study extended these findings to martensitic stainless steel, offering further insights into the chemical evolution of passive films under electrochemical stress [67–68].

#### Batteries

The dip-and-pull method allows for surface-sensitive XPS measurements of interfaces that are typically hidden inside batteries. Whilst the cell geometry obviously differs dramatically from that of any conventional cell, the approach can provide a valuable model system to study fundamental chemical processes. Experiments can take two forms. First, dip-and-pull can be used to measure a “thick” film (greater than the ~10 nm information depth of XPS) of electrolyte solution. Kallquist et al. have shown that measurements of these thick liquid films can be used to infer charge transfer processes, since the emitted kinetic energy of a photoelectron is dependent on the potential of the probed liquid relative to the grounded working electrode [69–70]. Specifically, the interfaces of gold, copper oxide, and LiNi_1/3_Mn_1/3_Co_1/3_O_2_ with 1 M LiClO_4_ in propylene carbonate have been studied. When there is no lithiation/delithiation, the expected 1:1 ratio of changes in photoelectron kinetic energy and applied potential are observed. However, when high enough potentials are reached to induce lithiation/delithiation, a deviation from this ratio is observed. These measurements demonstrate how APXPS, through its sensitivity to potential changes, can be used to probe interfacial chemistry without direct access to the interface.

**Figure 15:**
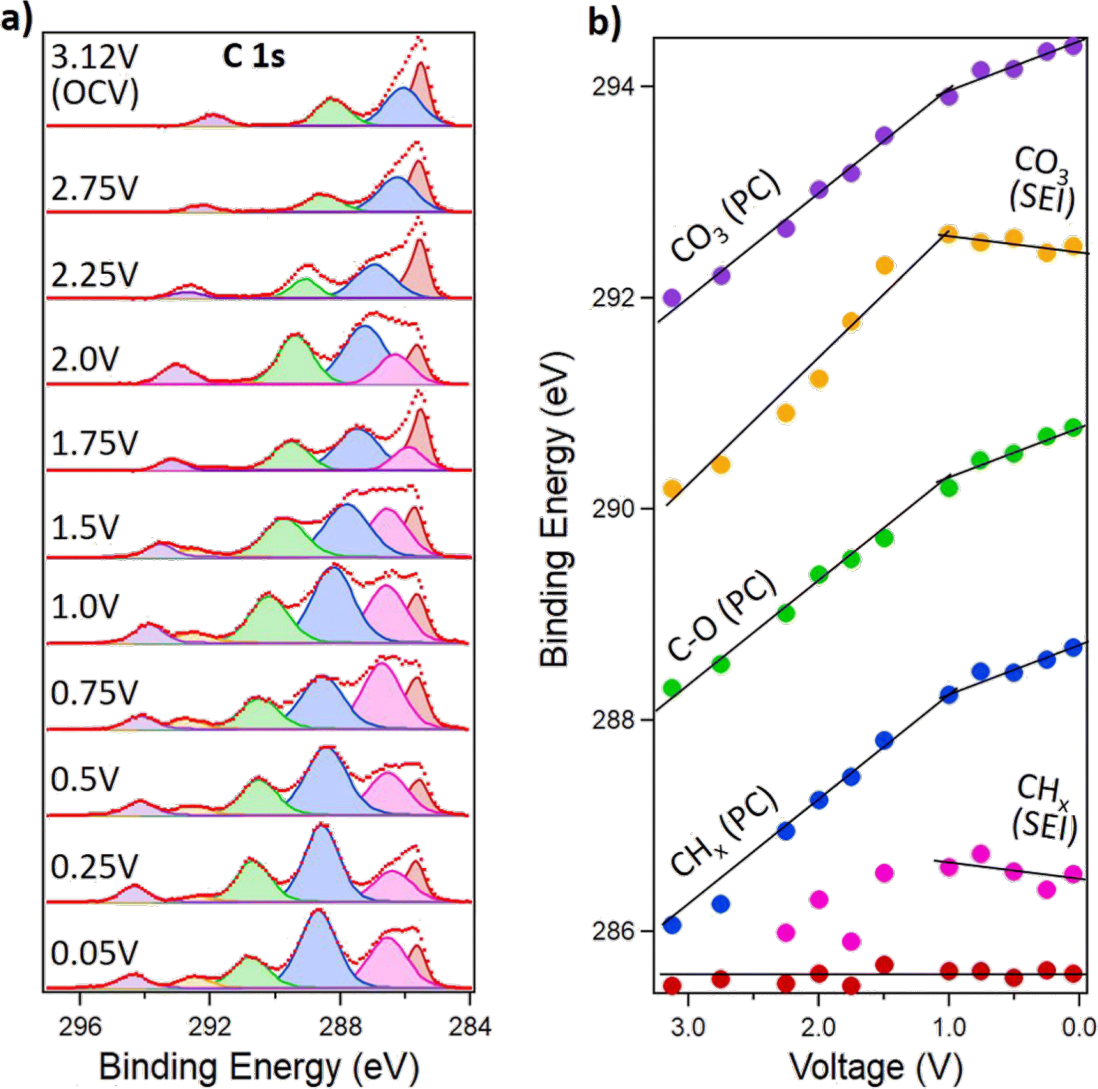
(a) C 1s spectra of the interface between glassy carbon and a 1 M LiPF_6_/propylene carbonate electrolyte as a function of cell potential. (b) Trends of the binding energies of the various spectral components. Figure 15 was reproduced from [71] with permission of the Royal Society of Chemistry. This content is not subject to CC BY 4.0.

The second form of experiment studies the solid–liquid interface directly, which, in the case of batteries, is where solid–electrolyte interphases (SEIs) form. Having stable SEIs formation is critical for the performance of a battery. A stable SEI stops the electrode from degrading, but uncontrolled growth can, in the extreme case, lead to short-circuit conditions inside the cell. Dip-and-pull XPS has been used to study the growth of SEIs at the interface between a 1 M LiPF_6_ in propylene carbonate electrolyte solution and a glassy carbon working electrode in a two-electrode cell with a Li metal counter electrode [71]. Here, XPS of the electrode–solution interface was measured at series of voltage steps. First, the electrodes were dipped into the solution, and the cell voltage was swept to the desired voltage. Then the electrode was pulled up under a potential hold for XPS measurements, and, due to the favorable wetting properties of this choice in materials, it was possible to find an area at the top of the liquid meniscus that was thin enough for photoelectrons generated at the electrode surface to escape and be measured. By repeating this dip-and-pull process at different potentials, the evolution of the carbonate and hydrocarbon SEI components could be observed (Figure 15). Similar studies have been performed on sputtered LiCoO_2_ cathodes, demonstrating the broader applicability of this technique to conventional battery materials [10].

## Conclusion and Perspectives

Ambient pressure X-ray photoelectron spectroscopy has emerged as a powerful and versatile technique for probing surface and interface phenomena under realistic environmental conditions. At MAX IV, the synergy between fourth-generation synchrotron source brightness and beamline-specific developments, such as advanced electron analyzers, tailored AP cells, and time-resolved detection schemes, has enabled operando and in situ investigations with unparalleled time resolution. These capabilities have pushed the boundaries of surface science, allowing for direct insights into reaction mechanisms in catalysis, electrochemistry, thin film growth, and corrosion.

The examples presented throughout this review, ranging from single-atom catalyst stabilization and confined 2D reactivity, to time-resolved studies of gas–solid reactions, photocatalytic water splitting, atomic layer deposition, and electrochemical interfaces, demonstrate the breadth of scientific questions that APXPS can now address. MAX IV’s SPECIES and HIPPIE beamlines have contributed to this development, not only by offering high-performance instrumentation but also by fostering integrated experimental strategies and user-driven innovation.

Looking ahead, one of the most significant challenges for the APXPS community is the transition from simplified model systems to realistic, complex materials operating under industrially relevant conditions. Achieving this shift will require further innovations in sample environments, pressure compatibility, and surface sensitivity, especially for systems that exhibit spatial or chemical heterogeneity at the micro- and the nanoscale.

A key direction for the future lies in the multimodal integration of APXPS with complementary surface-sensitive and bulk-sensitive techniques. Combining APXPS with vibrational spectroscopies (such as IRRAS and Raman), structural probes (such as X-ray absorption spectroscopy or X-ray diffraction), and mass spectrometry will enable a more holistic understanding of structure–function relationships at working interfaces. Additionally, the development of correlated, spatially resolved measurements, potentially via photoemission electron microscopy (PEEM) or scanning probe methods, could further bridge the gap between fundamental surface studies and application-relevant complexity.

Another promising frontier is the coupling of APXPS with advanced data acquisition and analysis, such as machine learning for spectral interpretation, real-time kinetic modeling, and high-throughput experimentation. These approaches will be instrumental in interpreting the large, multidimensional datasets produced by time-resolved and pressure-dependent measurements, especially in dynamic systems with multiple reaction pathways.

Ultimately, the goal is to move beyond the use of APXPS as a purely observational tool and to establish it as a quantitative, mechanistic probe that directly informs materials design and process optimization. By continuing to refine its resolution, sensitivity, and integration with complementary modalities, APXPS is positioned to contribute to pressing societal challenges, including clean energy conversion, sustainable catalysis, environmental remediation, and next-generation electronic and electrochemical devices.

Through its unique ability to reveal buried, reactive, and dynamic interfaces under realistic conditions, APXPS will not only remain a cornerstone of modern surface science, but increasingly, a bridge between fundamental understanding and functional performance.

## Data Availability

Data reported in Figures 6, 7 and 8 are new and never published.
